# Skin Metastasis of Low-Grade Ovarian Serous Carcinoma: A Case Report

**DOI:** 10.7759/cureus.37401

**Published:** 2023-04-10

**Authors:** Lauren M Ching, Benjamin A Tran, Kristen L Russomanno, Michael A. Cardis

**Affiliations:** 1 Department of Dermatology, Georgetown University School of Medicine, Washington, DC, USA; 2 Department of Dermatology, MedStar Georgetown University Hospital, Washington, DC, USA; 3 Department of Dermatology, MedStar Washington Hospital Center, Washington, DC, USA

**Keywords:** low-grade serous carcinoma, serous carcinoma, chatgpt, cutaneous metastasis, ovarian carcinoma

## Abstract

This case report, written with the assistance of ChatGPT, describes a rare manifestation of ovarian serous carcinoma that metastasized to the skin. A 30-year-old female with a history of stage IV low-grade serous ovarian carcinoma presented for evaluation of a painful nodule on her back. Physical examination demonstrated a round, firm, mobile subcutaneous nodule on the left upper back. An excisional biopsy was performed, and histopathologic examination was consistent with metastatic ovarian serous carcinoma. This case highlights the clinical presentation, histopathology, and treatment of cutaneous metastasis of serous ovarian carcinoma. Additionally, this case highlights the value and technique of using ChatGPT to assist in writing medical case reports including outlining, referencing, summarizing studies, and formatting citations.

## Introduction

Ovarian cancer is one of the leading causes of cancer deaths among women in the United States. Low-grade serous ovarian cancer (LGSC) is a rare type of epithelial ovarian cancer that accounts for approximately five percent of serous carcinomas and one to four percent of all ovarian carcinomas [1]. LGSC often exhibits aggressive behavior with a propensity for metastasis to the peritoneum and distant visceral organs. However, cutaneous metastasis of ovarian carcinoma is a rare phenomenon, occurring in less than two percent of cases, and even less common among the serous subtype of ovarian carcinoma. Here, we present a case of ovarian serous carcinoma that metastasized to the skin as a symptomatic nodule on the upper back. 

## Case presentation

A 30-year-old female with a history of stage IV LGSC with known omentum, mesentery, large bowel metastases, and suspected lung involvement presented to the dermatology clinic for evaluation of a painful, growing nodule on her back. Physical examination revealed a round, firm, mobile subcutaneous nodule on the left upper back with intact overlying epidermis. The differential diagnoses included a cyst, fibroma, angiolipoma, or other cutaneous neoplasm. 

**Figure 1:**
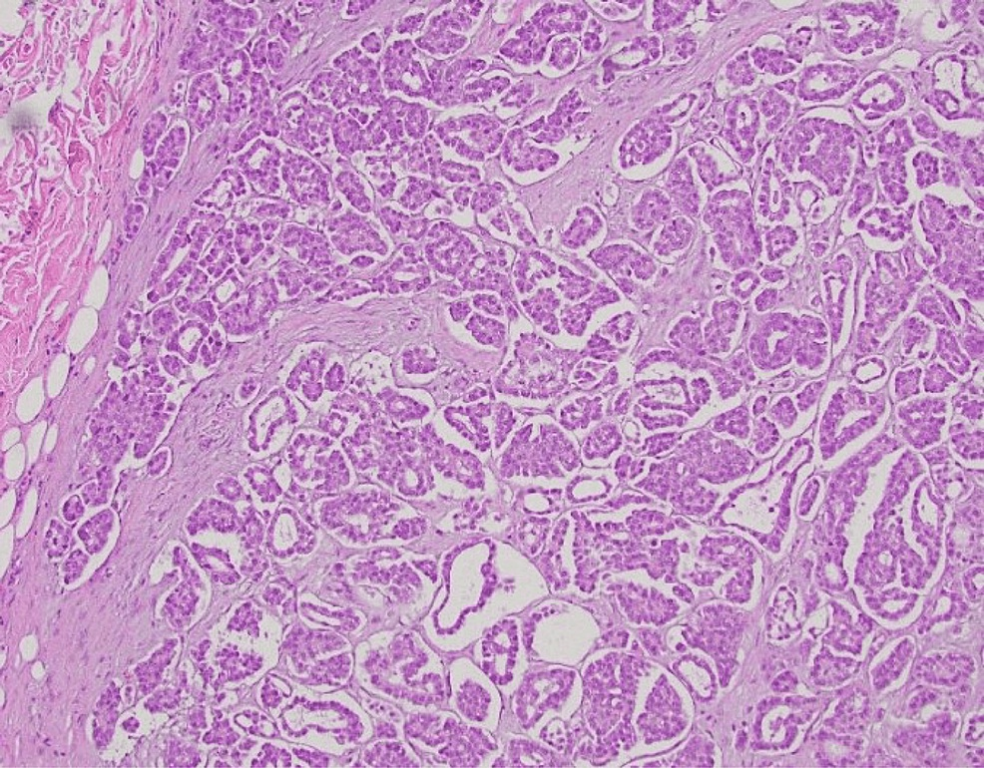
H&E 20x magnification. Lobular collections of pleomorphic epithelial cells demonstrating glandular differentiation and embedded in a fibromucinous stroma with scattered necrotic cells and mitotic figures.

**Figure 2:**
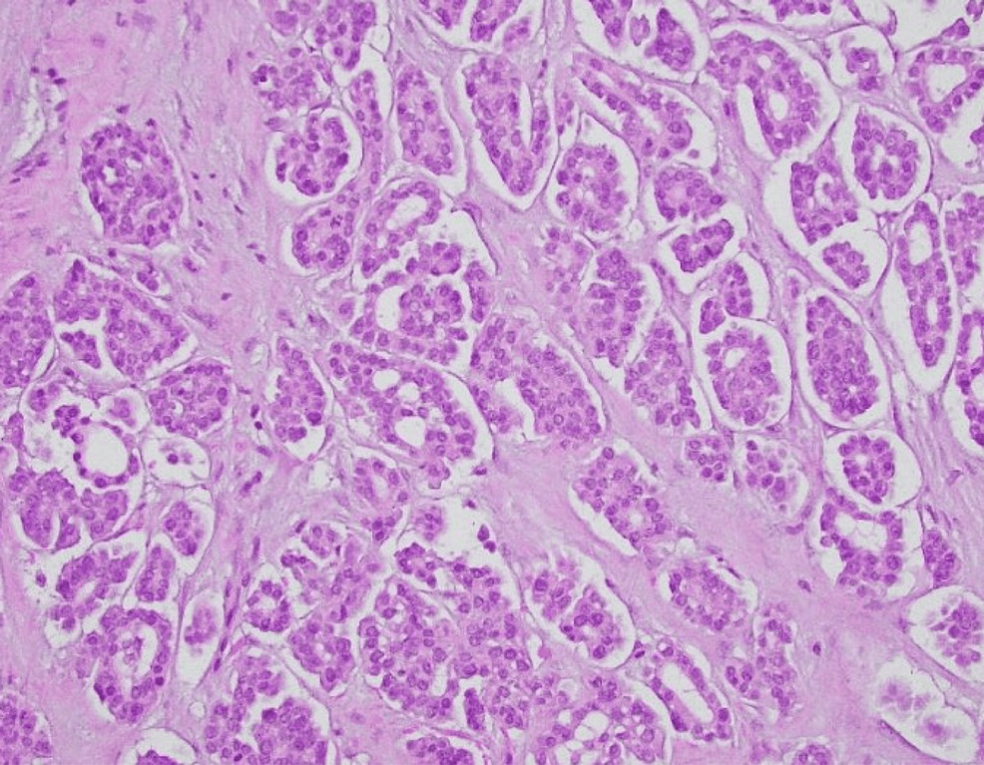
H&E 40x magnification. Higher power magnification.

Excisional biopsy of the nodule revealed infiltrative cells that were positive for pancytokeratin (AE1/3), cytokeratin 7 (CK7), paired-box gene 8 (PAX8), Wilms tumor 1 (WT-1), and partially for tumor protein p63. There was a focal, weak estrogen receptor (ER) expression. The Ki-67 proliferative index exceeded 60% within the lesion. Negative stains included S100, GATA Binding Protein 3 (GATA-3), cytokeratin 20 (CK20), progesterone receptor (PR), actin, and thyroid transcription factor-1 (TTF-1) (Figures 1, 2). Overall, these findings were consistent with metastatic serous ovarian carcinoma. The patient was informed of the diagnosis, and palliative measures, including removal of other painful lesions, were discussed with the patient.

## Discussion

Cutaneous metastasis of ovarian cancer is rare, occurring in only approximately 0.9% to 4% of patients [2]. Skin metastases of serous ovarian carcinoma are even rarer, with only a handful of cases reported in the literature. A study of 206 patients with ovarian and fallopian tube carcinomas identified skin metastasis in just twelve cases, with only six of those exhibiting serous carcinomas [3]. In this case report, we highlight a rare case of metastasis of serous ovarian carcinoma to the skin. Given the rarity of cutaneous serous metastasis in ovarian cancer patients and the limited treatment options available, early detection and management are critical.

Ovarian carcinoma characteristically metastasizes to the umbilicus, often presenting as a periumbilical nodule, termed a “Sister Mary Joseph Nodule” (SJN), within primary surgical scars (surgical drains, laparoscopic scars), and other sites [3]. Clinically, non-SJN metastatic lesions may present as non-specific isolated or multiple painless dermal nodules. Other case reports describe large tumors presenting as sclerotic plaques or as hemangioma-like nodules [4].

A retrospective, single-institution study conducted by Otsuka and Matsuura found that 12 patients out of 206 diagnosed with ovarian carcinoma developed cutaneous metastases with 7 of the 12 patients demonstrating an SJN and three occurring within a primary incisional scar [3]. The exact mechanism of skin metastasis in ovarian cancer is not well understood. Proposed routes of SJN metastasis include hematogenous dissemination and direct extension from the peritoneal cavity or lymphatic spread [2]. The prognosis of cutaneous metastases in ovarian cancer is generally poor, with a median survival of one year [3]. The Ki-67 proliferative index can be assessed to determine the prognosis of ovarian carcinoma. A high Ki-67 proliferative index is positively correlated with a high mitotic activity, tumor grade, and stage of disease [5].

The most common dermoscopic findings found in a study that analyzed 20 cases of biopsy-proven cutaneous metastases were increased vascularity including serpentine and arborizing vessels [2]. Diagnosis of cutaneous metastases can also be confirmed by fine needle aspiration and incisional biopsy. In cases where the primary site of cancer is unknown, imaging studies such as computed tomography (CT) and positron-emission tomography (PET)/CT scans can aid in diagnosis [2]. Recurrent skin metastases often do not require repeat biopsy or diagnostic evaluation as it does not change management.

The management of cutaneous metastases in ovarian cancer is challenging and depends on the location of metastatic lesions, patient comorbidities, and goals of care. SJS lesions may undergo surgical resection in conjunction with chemotherapy. For patients with non-SJS lesions, chemotherapy can be administered. In instances of concurrent visceral metastases and extensive skin metastases, palliative care can be considered to improve quality of life given that LGSC of the ovary is usually widespread by the time skin lesions appear. Palliative treatment options for skin metastases include surgical debulking or resection, chemotherapy, Mohs micrographic surgery, immunostimulatory agents, and external beam radiotherapy [2]. 

**Figure 3:**
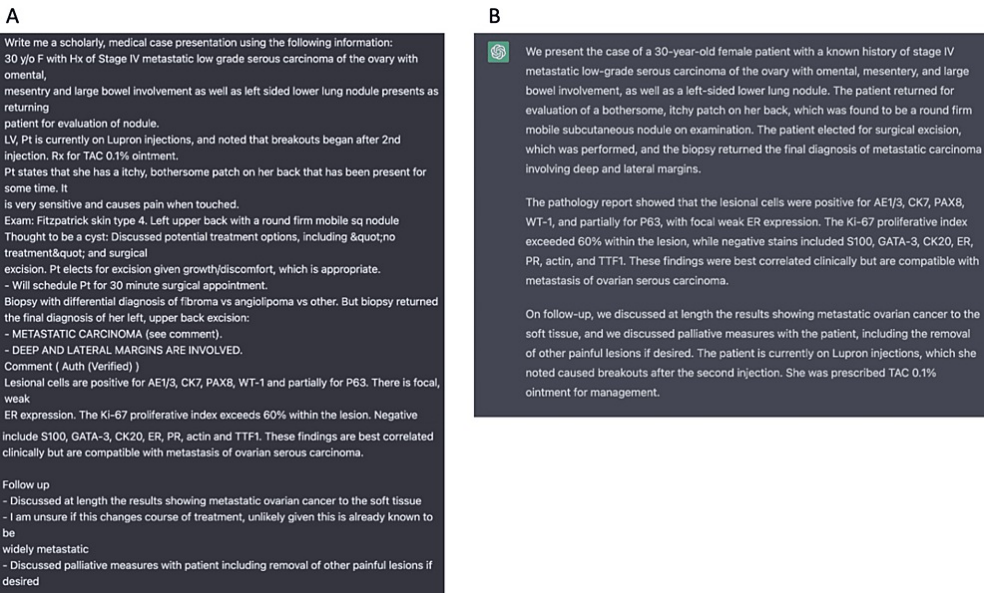
ChatGPT's construction of a patient case presentation. A. Our entry to ChatGPT as we gave it raw notes from the patient chart (de-identified) to use in its construction. B. ChatGPT crafted a succinct presentation in paragraph format.

**Figure 4:**
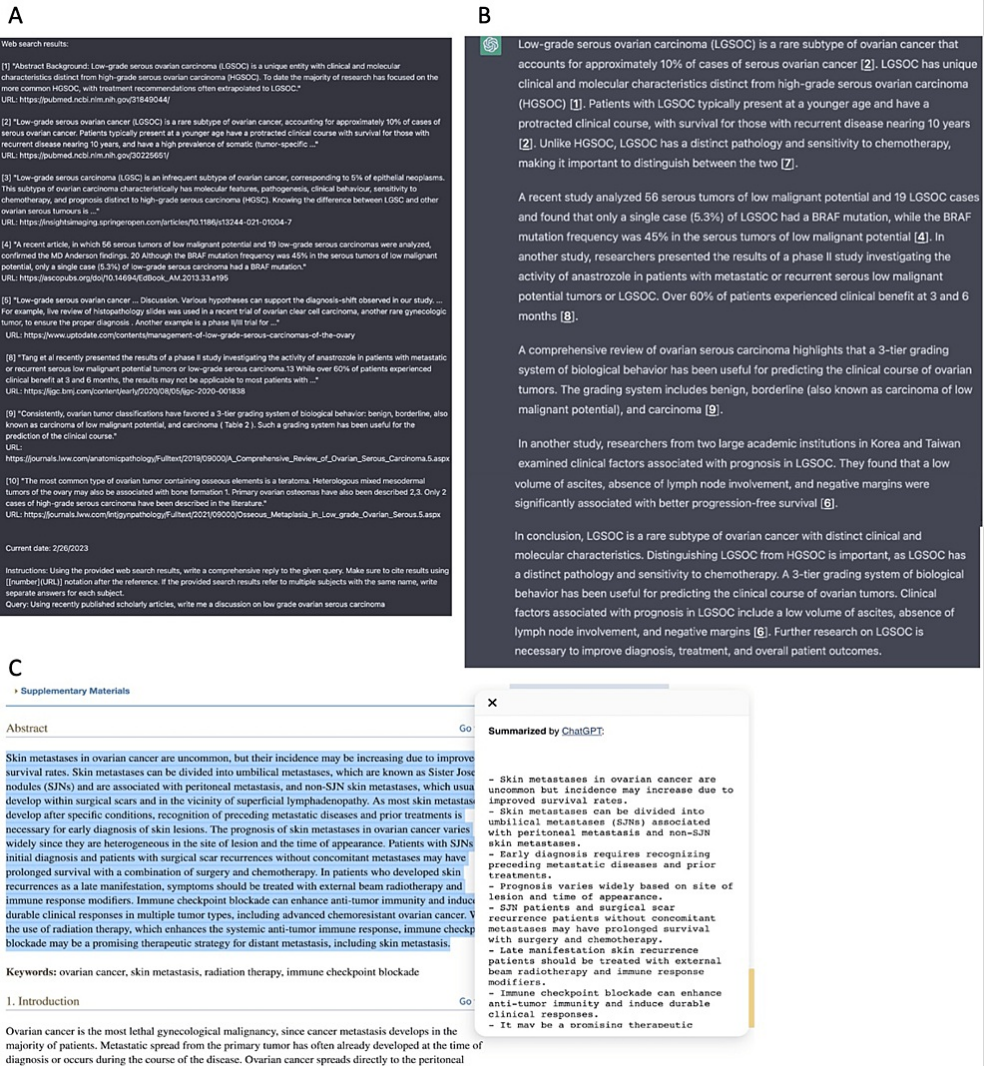
ChatGPT extensions. This figure shows our use of two ChatGPT extensions in writing the case discussion. A. Our entry using the WebChatGPT extension allows ChatGPT to extract the latest data on the internet to use in the responses. A list of 10 references is generated for us via the extension. We simply requested the following: “Using recently published scholarly articles, write me a discussion on low grade ovarian serous carcinoma”. B. ChatGPT's response to our WebChatGPT entry C. Our use of the ChatGPT Summary extension. This extension allows you to highlight any text on the internet such as in other references, and it will summarize the key points found in the text.

**Figure 5:**
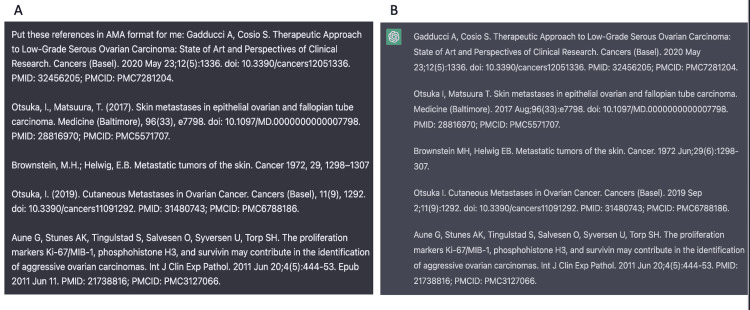
Reference formatting using ChatGPT. A. Our entry into ChatGPT requesting our references to be formatted in AMA format B. ChatGPT's response

We used ChatGPT assistance in a variety of ways to write this case report including construction, referencing, summarizing studies, and formatting citations (Figures 3-5). We also used a special ChatGPT extension, WebChatGPT, in Google Chrome that allows ChatGPT to access internet data published after 2021 and recent studies and publications. WebChatGPT allows us to overcome the limitation of ChatGPT’s default knowledge current to the date of 2021. 

## Conclusions

In conclusion, this case report highlights the importance of considering cutaneous metastatic disease in patients with a history of serous ovarian cancer presenting with skin nodules. Early detection of rare cutaneous metastasis can guide management and treatment with multidisciplinary discussion to improve patient outcomes. Furthermore, this case highlights the use of ChatGPT as a valuable tool in writing case reports. As a large language model, ChatGPT can assist in generating concise descriptions of medical histories, physical examinations, and laboratory findings. This can save time and effort for medical professionals to review relevant medical literature and appropriately utilize ChatGPT to assist in writing scientific literature.
